# The Past and Present of Dentistry

**Published:** 1870-05

**Authors:** Benj. F. Smith

**Affiliations:** New Orleans


### THE PAST AND PRESENT OF DENTISTRY.

BY DR. BENJ. F. SMITH, OF NEW ORLEANS.

Read before the Southern Dental Association.

Mr. President and Gentlemen:—I have listened with pleasure to remarks illustrating the wonderful progress of Dental art and science since I came upon the stage. I think we have reason to be proud of our noble profession, which I regret to hear some of our members designate as an "humble profession." That it was exceedingly humble when I graduated thirty years since, in the then little town of Erie, Pa., I freely admit—you are startled at the statement that there was a Dental school at that early day, and I have no doubt you will claim for Baltimore the credit of the first Dental College. You are, no doubt, proud that you can boast of several Dental Colleges, each with eight or ten professors, where aspiring youths are required to study three years before they are entitled to write D. D. S. after their names. Not so in my day. We had but one Professor, and he was a man of such varied parts that he made a first-class Dentist of me in ten days. I see a smile of incredulity. If any one is so bold as to doubt my word I will produce his certificate that I was duly prepared to practice the art and mystery of Dentistry. Gentlemen, that was the day of secrets and mysteries. In the course of my early career I purchased several, and I held on to them with great tenacity. In fact this was the only way we could show our superiority over the *quacks* that were then beginning to flood the country. But, gentlemen, if you are still incredulous, I will add another proof. If you will listen to me for a few minutes, I promise to repeat all he ever taught me. I have still another. You all recognize Professor Atkinson as the foremost scientific and practical Dental surgeon in the world. He, sir, graduated in that kind of a school, and we commenced our practice together in the section bordering on

Lake Erie. I will add another, and I think, a most convincing proof of my superior qualifications, and the claims of my *alma mater* for pre-eminence. In my course of preparation I had acquired an extensive list of high-sounding Latin names, and the way I slung them out was very astonishing to the good people for whom I condescended to operate. I well remember the proud satisfaction with which I used such words as *Dens Sapientia*, *Central Incisors*, *Bi-Cusped*, *Superior Cuspidatus*, *Ligamentum Dentis*, etc. Another proof that I fully availed myself of the superior advantages of that early day, may be inferred from the fact that I at once obtained an extensive practice, and in less than six months I had whole stacks of certificates from M. Ds., D. Ds., and Professors of Colleges, stating that I was the most skillful and accomplished Dental Surgeon they had ever known. True, many had seen but one full-fledged Dentist before, and he traveled through the South, where he had made so much money that his neighbors shrewdly suspected that he must have added the profession of counterfeiting to that of Dental surgery, or he could not have purchased his splendid farm so quickly. I will add one more proof, which, I think, will satisfy you that my ten days collegiate course had fully qualified me for the wonder and admiration of those honest people, who were justly astonished that one small head could contain such an amount of scientific and linguistic lore, and whose hands possessed such an amount of cunning and skill. I actually manufactured several Dentists myself in an equally short space of time, and I can truthfully say, that they were fully as well prepared as myself to astonish other natives with their learning and skill.

I would like to compare some of the present methods of practice, with those of that day. There sits my worthy and venerable friend, Dr. F. H. Knapp, a veteran of thirty-five years practice, to whom belongs the distinguished honor of introducing the mallet for filling teeth; and I, gentlemen, have the honor of being his first patient, or victim if you please. I well remember the first complete set of instruments I ever saw, which cost the Doctor the large sum of $8. His stock for filling teeth consisted of about one pound of block tin; this he formed into plugs with his jack-knife, and with mallet and punch he drove them into my molars. To supply himself with teeth, he made a raid into a butcher's yard, and drew them from the lower jaw of a calf that had met an untimely end.

You are justly proud of the two Dental chairs that have been on exhibition. They are certainly marvels of ingenuity and skill, elegance and comfort. You must not ignore, however, the chair of my day. It certainly had the merit of simplicity and cheapness, and was all that we then desired. It consisted of two common chairs. In the one we placed the patient, and on the other one foot; our knee formed a most inconvenient head-rest. I soon made a notable improvement on this, and only missed a fortune by neglecting to patent this marvel of ingenuity and simplicity. I used a common wash-tub, on which I placed a rocking-chair. By sliding the chair back and forth, I secured the desired position. You can not fail, gentlemen, to give me credit for the profound discovery that the said wash-tub had two ears, and that the perforations through those ears were most perfectly adapted to the binding of the aforesaid chair to the said tub, and thus assuring a substantial basis for the impending operations. This would, unquestionably have entitled me to a patent.

I will next allude to, or compare the present with the prices of the good old days when everybody was honest. We had wholesale and retail prices. A single filling of tin was 50c. at retail, and 25c. at wholesale, and gold fillings were double that price.

Amalgam fillings were introduced about that time by the celebrated Crawcours I purchased the secret, and presently acquired great skill in the plastering art. I also made the profound discovery that from ten to twenty fillings would sometimes produce salivation.

In 1843 I settled in New Orleans, and after many misgivings, adopted the then current price, three dollars for an ordinary filling. My friend, Dr. Atkinson, learning that I was charging this extortionate price very naturally concluded that I must have left my conscience at the North But times change and we change with them. On a visit to New York last summer, I was delighted to meet my old time friend, who soon initiated me into the modern system of malleting and charging by inserting for me one filling, for which his regular charge is $500, and three others at from $80 to $120 each. I was slightly startled at such a giant stride in progress. But there was no ground for questioning the fact, and I was seriously assured that it was directly sanctioned by Angelic Inspiration. I shall not be so bold as to deny the proposition, but I shall affirm that Dr. A. is the prince of good fellows, a thoroughly educated scholar and scientist, and the originator of many valuable improvements, and richly deserves the gratitude of the entire profession, and the extraordinary fees his wealthy patients are able to pay him.

I will close by offering two pieces of advice:

First—Do not attempt to conform your fee bills to those first mentioned. You can not hope to succeed. Leave us alone in our proud preëminence. We lived in a day when the "auri sacra fames," the infernal thirst for gold, had not permeated the entire social mass.

Second—I can not conscientiously advise you to imitate our illustrious friend, Dr. A., in estimating the value of your labor and gold at $1920 per ounce. No, not even if you are so fortunate as to secure the constant attendance of two celestial advisers. I can not seriously advise you to surrender your judgment and manhood to the dictum of some unseen, intangible presence. How can you know but the ghost of some departed rival Dentist is bent upon doing you mischief, and appears for this purpose in the garb of a friend and angel of light. He may flatter your vanity, and tickle your fancy with the idea that you are made of so much finer materials than ordinary mortals that you are permitted to hold communion with the progressed in other spheres. Your particular angel may prove a fallen one, who has assumed the role of mentor and adviser, that he may work your injury by inducing you to assume the ridiculous position of being run by invisible engineers from the Great Beyond.

